# Oral mucosa - an examination map for confocal laser endomicroscopy within the oral cavity: an experimental clinical study

**DOI:** 10.1007/s00784-024-05664-9

**Published:** 2024-04-23

**Authors:** Nicolai Oetter, Jonas Pröll, Matti Sievert, Miguel Goncalves, Maximilian Rohde, Christopher-Philipp Nobis, Christian Knipfer, Marc Aubreville, Zhaoya Pan, Katharina Breininger, Andreas Maier, Marco Kesting, Florian Stelzle

**Affiliations:** 1 Department of Oral and Maxillofacial Surgery, Friedrich‑Alexander University Erlangen‑Nürnberg (FAU), University Hospital Erlangen, Glückstraße 11, 91054 Erlangen, Germany; 2 SAOT‑Erlangen Graduate School in Advanced Optical Technologies, Friedrich‑Alexander University Erlangen‑Nürnberg (FAU), Paul Gordan Straße 6, 91052 Erlangen, Germany; 3 Department of Otorhinolaryngology, Head and Neck Surgery, Friedrich‑Alexander University Erlangen‑Nürnberg (FAU), University Hospital Erlangen, Waldstraße 1, 91054 Erlangen, Germany; 4 Department of Otorhinolaryngology, Head and Neck Surgery, Julius-Maximilians University Würzburg, University Hospital Würzburg, Josef-Schneider-Straße 11, 97080 Würzburg, Germany; 5 Department of Oral and Maxillofacial Surgery, University Hamburg, University Medical Center Hamburg- Eppendorf, Martinistraße 52, 20246 Hamburg, Germany; 6 Technische Hochschule Ingolstadt, Esplanade 10, 85049 Ingolstadt, Germany; 7 Pattern Recognition Lab, Department of Computer Science, Friedrich‑Alexander University Erlangen‑Nürnberg (FAU), Martensstraße 3, 91058 Erlangen, Germany; 8 Department Artificial Intelligence in Biomedical Engineering, Friedrich‑Alexander University Erlangen‑Nürnberg (FAU), Henkestraße 91, 91052 Erlangen, Germany

**Keywords:** Confocal laser endomicroscopy, Oral cavity, Oral carcinoma, Optical methods, Oral cavity squamous cell carcinoma, Head and neck cancer

## Abstract

**Objectives:**

Confocal laser endomicroscopy (CLE) is an optical method that enables microscopic visualization of oral mucosa. Previous studies have shown that it is possible to differentiate between physiological and malignant oral mucosa. However, differences in mucosal architecture were not taken into account. The objective was to map the different oral mucosal morphologies and to establish a “CLE map” of physiological mucosa as baseline for further application of this powerful technology.

**Materials and methods:**

The CLE database consisted of 27 patients. The following spots were examined: (1) upper lip (intraoral) (2) alveolar ridge (3) lateral tongue (4) floor of the mouth (5) hard palate (6) intercalary line. All sequences were examined by two CLE experts for morphological differences and video quality.

**Results:**

Analysis revealed clear differences in image quality and possibility of depicting tissue morphologies between the various localizations of oral mucosa: imaging of the alveolar ridge and hard palate showed visually most discriminative tissue morphology. Labial mucosa was also visualized well using CLE. Here, typical morphological features such as uniform cells with regular intercellular gaps and vessels could be clearly depicted. Image generation and evaluation was particularly difficult in the area of the buccal mucosa, the lateral tongue and the floor of the mouth.

**Conclusion:**

A physiological “CLE map” for the entire oral cavity could be created for the first time.

**Clinical relevance:**

This will make it possible to take into account the existing physiological morphological features when differentiating between normal mucosa and oral squamous cell carcinoma in future work.

**Supplementary Information:**

The online version contains supplementary material available at 10.1007/s00784-024-05664-9.

## Introduction

Head and neck squamous cell carcinomas (HNSCCs) are world’s sixth most common cancer [1]. The heterogeneous group of (head and neck) cancers affects more than 2.5 million people and causes 379,000 deaths per year [2, 3], mainly caused by the consumption of tobacco and alcohol [4].

Currently, invasive tissue biopsy is still the gold standard for confirming a (suspected) diagnosis, regardless of whether it is a benign or malign mucosal lesion [5–7]. Because of the resulting risks of invasive interventions to confirm the diagnosis (e.g., bleeding and infections [8]) non-invasive techniques have gathered more attention during the last years. Non-invasive optical methods (e.g. reflectance confocal microscopy) have already been successfully tested within the oral cavity for hard tissues (e.g. tooth enamel) and delivered promising results in preliminary studies [9]. These optical methods, e.g. confocal laser endomicroscopy (CLE), can also be used to examine oral soft tissues.

CLE has been used since 2006 [10] and increased its importance constantly, especially in (non- and minimal-invasive) imaging of the upper and lower gastrointestinal tract [11]. It can be used to visualize the mucous membranes (in vivo) with a thousand-fold magnification in real time. This technique has been investigated with encouraging results for HNSCCs [12, 13] including the development of a classification and scoring system to facilitate assessment of CLE sequences in head and neck surgery [12, 13]. Here [12, 14] it was shown that CLE can be used to differentiate between physiological oral mucosa and oral squamous cell carcinoma. Sensitivity (range from 0.901 to 0.973) and specificity (range from 0.874 to 0.889) of the diagnoses by CLE experienced as well as inexperienced examiners correlated well with the results of tissue histopathology.

However, it is known that the oral cavity itself has a large and complex heterogeneity of epithelial and cellular architecture [15, 16] and these preliminary studies did not (sufficiently) differentiate between CLE imaging of the different regions within the oral cavity and its influence on CLE image quality and informative value. A differentiation of malignant versus physiological tissue is only possible to a very limited extent without this knowledge.

It can be assumed that, in addition to clinical experience and subjectivity inherent in the evaluation of CLE images [17], the heterogeneous epithelial and subepithelial architecture of the oral cavity affects CLE imaging results.

In this study, the suitability of the CLE examination for the various localizations within the oral cavity was investigated and a CLE map of the physiological oral mucosa was created for the first time. Knowledge of natural morphological variability forms the basis for a reliable differentiation between carcinoma and physiological mucosa in all regions of the oral cavity and is therefore an essential initial step.

## Materials and methods

### Study design and approval by ethics committee

This experimental clinical study was carried out to create a CLE map of physiological oral mucosa. Phase 1 consisted of CLE imaging (data acquisition). Phase 2 was the image analysis, interpretation and (statistical) evaluation. The study was supported by the German Research Foundation (Number 439,264,659) and was approved by the ethics committee of the University of Erlangen-Nürnberg (number: 243_12 B). It respected the principles of the ethics committee in charge as well as the 1975⁄1983 Helsinki declaration.

### Patients selection, inclusion and exclusion criteria

In total, our study includes twenty-seven patients who visited the Oral and Maxillofacial Surgery Department of the Erlangen University Hospital (Germany) for further examination of suspect lesions of their oral cavity (*n* = 27). These patients were suspected of having a (malignant) degeneration of the oral mucosa or were presented to us to rule out a malignant disease (e.g. with a referral from a dental/medical colleague in a private practice). All patients had only one suspicious mucosal change (no multiple or diffuse lesions of the oral cavity). Before obtaining a tissue sample in the area of the suspicious mucosal lesion (under local anesthesia), the whole oral cavity of the patient was examined using CLE. Therefore, in addition to the suspect mucosal areas (subject of other studies), clinically unsuspicious/physiological mucosal areas of the oral cavity were also measured with the help of CLE. Only the physiological mucosal areas are the subject of this study. No tissue samples were taken from the clinically unsuspicious oral mucosa (for ethical reasons).

All of the above-mentioned patients who wanted to participate in the study were included consecutively. They were informed about potential risks and signed an informed consent form prior to examinations.

Exclusion criteria were an age < 18 years, a current pregnancy/lactation period, known allergies to the used contrast agent, renal insufficiency and beta-blocker medication.

### Image acquisition protocol and image analysis

Prior to the examination every patient received an initial intravenous injection of 3 milliliters of fluorescein (Fluorescein Alcon 10%).

Probe-based confocal laser endomicroscopy images were obtained 5–15 min after injection of fluorescein using a stand-alone probe-based CLE system (pCLE: ColoFlex UHD Probe, CellVizio, Mauna Kea Technologies, Paris, France). The system offers a video frame rate of 8 frames/s with a resolution of approx. 0.41 microns/pixel and a maximum penetration depth of 65 μm, allowing the cell structure and tissue architecture to be visualized. The diagnostic laser system used has a wavelength of 488 nm and therefore has no influence on the surface quality of the examined tissue (non-cutting). The examinations were performed by an examiner with experience of more than 50 patients in performing and interpreting CLE (head and neck area and gastrointestinal area) with Cellvizio certification (Mauna Kea Technologies). The measurement procedure was documented in a protocol (available as supplementary material). Examinations were taken from awake patients (chair-side).

The probe tip was placed in the oral cavity in the following order: Intraoral labial region of the upper lip (envelope fold), upper alveolar process, lateral border of the tongue, floor of the mouth (sublingual), hard palate and buccal site in the region of the intercalary line. At the end, the suspicious mucosal lesion was examined (not subject of the present study).

The resulting video sequences were inspected and analyzed independently by two CLE experts familiar with its use in the head and neck area and discussed in detail afterwards. The known parameters of preliminary studies [12, 14], such as tissue architecture, i.e. tissue homogeneity and intercellular gaps, cell morphology, fluorescence leakage and vessel morphology, were taken into account here. In addition, the image data was examined taking into account the following technical aspects, based on which quality and usability of the images were determined: Sharpness of the structures, noise-free images and number of artifacts (due to movement, mucus or incomplete surface contact of the probe).

### Statistical methods

Descriptive statistics of the demographic data of the included patients were performed using SPSS version 28 (IBM, Armonk, NY, USA).

## Results

The present video database was created based on measurements on twenty-seven patients, with eight female and nineteen male patients being examined. The mean age was 59.9 years (SD = 11.8, min. age = 40, max. age = 85). A total of 473 video sequences of the physiological oral mucosa were generated from the twenty-seven patients, which were of sufficient quality to be used for the analysis. Among the recorded data, there were no sequences that were not considered at all due to pronounced artifacts. The database reviewed and analyzed thus consisted of 5022 s (8 frames per second each) of video footage of physiological mucosa.

**Fig. 1:**
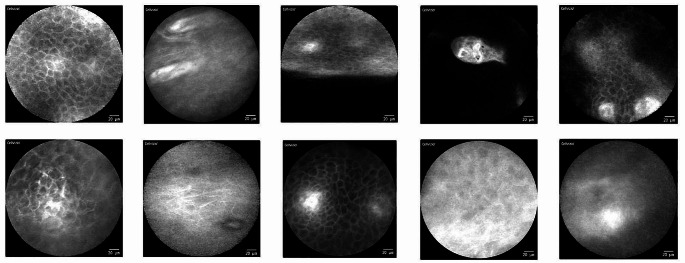
CLE imaging of physiological mucosa at different localizations of the oral cavity (examples): There is a very broad spectrum of different qualities and usability of the image data

The CLE videos/images show a very broad variety of quality and usability even though they were all taken by the same examiner with the same device (see Fig. 1).

**Fig. 2:**
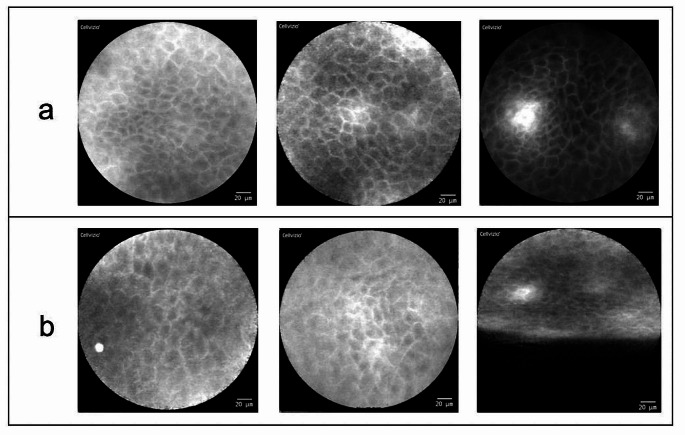
CLE imaging of the upper alveolar ridge **(a)** and the hard palate **(b)** (examples of three different patients): Physiological mucosa (attached gingiva) with completely organized tissue architecture: Slim and accurate intercellular gaps (light stripes) between every single cell (darker spots). Consistent cell morphology in shape and color with no amplified fluorescein leakage in between; regular vessel morphology (*bright white spot in the upper right and the lower left image*). Artifact at the lower edge of the lower right image (probe without full contact to the tissue)

The best results were recorded by imaging of the **upper alveolar process**. In this localization, usable image data could be acquired from each individual patient with a high degree of reliability. All these image data show the aspects of physiological oral mucosa (within CLE examination), which were already defined in preliminary studies [12]: homogeneous visual appearance with completely organized tissue architecture and slim, accurate intercellular gaps. Additionally, consistent cell morphology in shape and color, no amplified fluorescein leakage and regular vessel morphology were observed (see Fig. 2).

Similar results were achieved in the area of the **hard palate** (also Fig. 2). Here the images also show uniform cell structures with clear cell borders in comparable quality to the upper alveolar process.

**Fig. 3:**
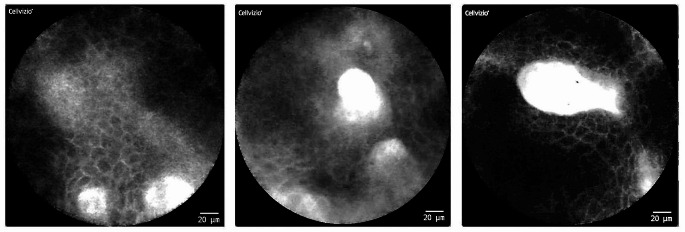
CLE imaging of the upper lip (intraoral; examples of three different patients): Physiological mucosa (lining mucosa) also with completely organized tissue architecture. Regular vessel morphology (bright white spots and loops) with increased occurrence compared to the other localizations

In the region of the **upper lip** (intraoral, envelope fold), there were more motion artifacts within the database compared to the other two localizations mentioned above. However, even here it was possible to obtain frames with good visualization of mucosal architecture. Overall, more blood vessels are visible here than at the other sites in the oral cavity. The images appear darker overall (see Fig. 3).

**Fig. 4:**
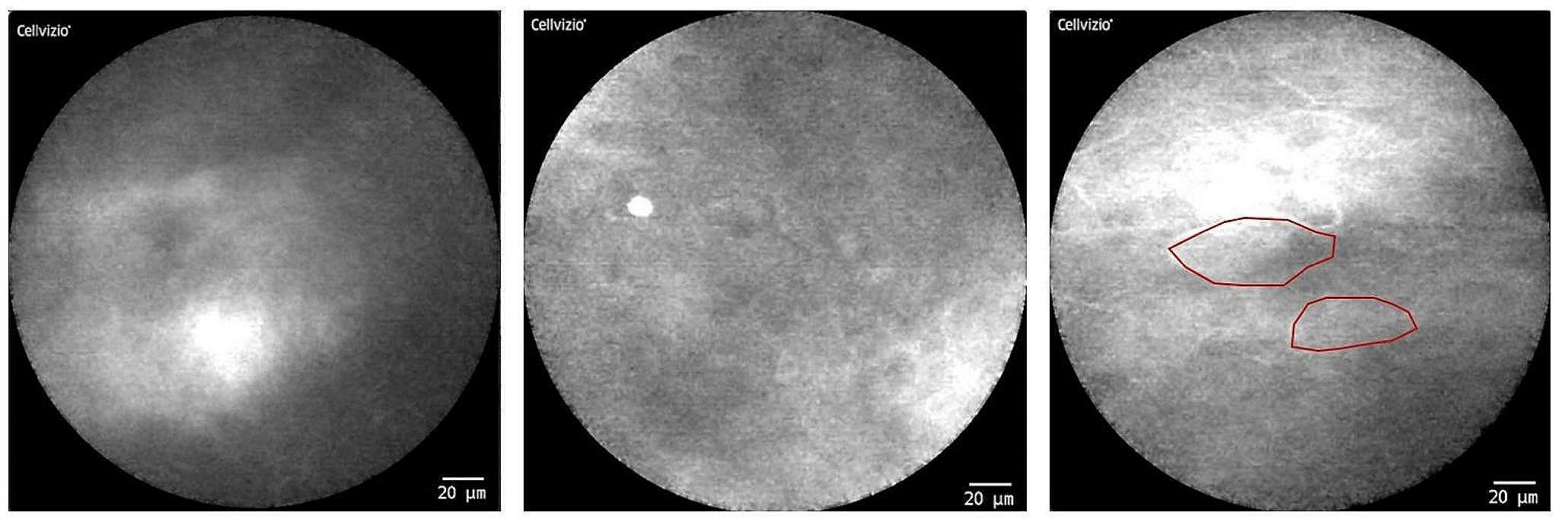
CLE imaging of the intercalary line (inside of cheek, examples of three different patients): Compared to the alveolar ridge, the hard palate and the inside of the lip, the individual cells are less clearly distinguishable from each other here. The intercellular gaps are less clearly visible. The overall appearance is “blurred and cloudy”. Isolated horn pearls (highlighted in red) may occur

Artifact-free imaging of the **buccal site in the intercalary line region** was difficult, but with patience and practice successful, so that the majority of the generated imaging data was usable for further evaluation. It is noticeable that in many CLE frames there are broad stripes or longitudinal oval shapes that appear to be horn pearls. The individual cells in this area tend to be less clearly distinguishable from each other and the intercellular gaps are less clearly visible. The overall appearance is rather “blurred and cloudy” (see Fig. 4).

**Fig. 5:**
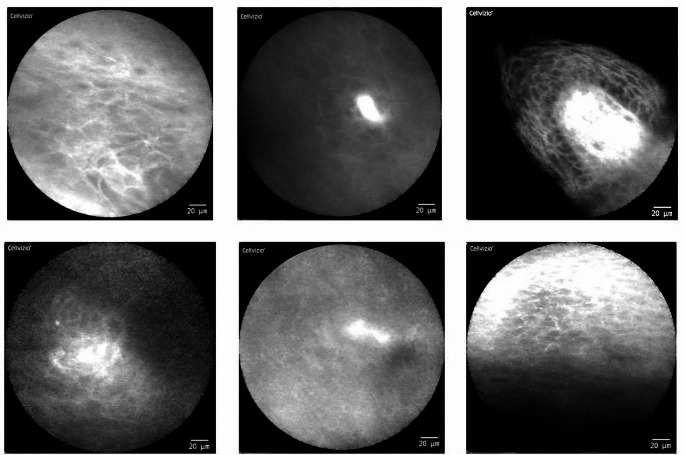
CLE imaging of the lateral tongue (example images): Overall, the conditions for generating sufficient image data are more difficult due to the increased movement of the tongue. There is a wide range of different image qualities and morphological conditions (from " cloudy appearance” to clearly visible cell structures)

**Fig. 6:**
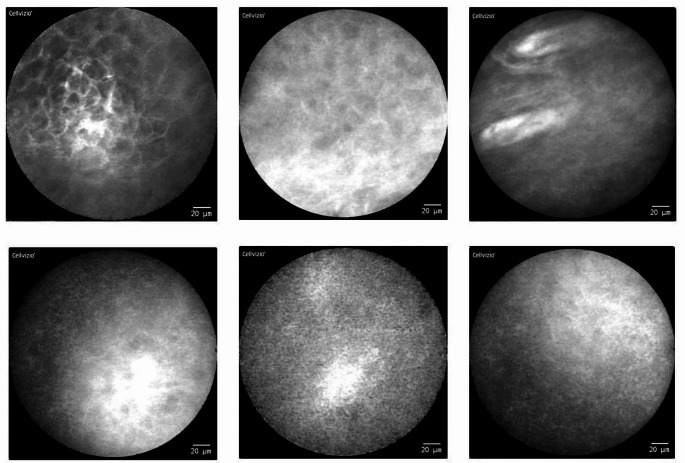
CLE imaging of the floor of the mouth (examples images): Similar to the tongue margin, the conditions for generating sufficient image data are also more difficult here (due to the presence of saliva). There is also a wide range of different image qualities and morphological conditions

Similar difficulties can be seen in the area of the **lateral edge of the tongue and the floor of the mouth**. Increased artifacts (movement and presence of saliva) occur here as well. Some CLE images of the lateral tongue were only partially filled with parts of the scanned region and could only be partially evaluated. However, the structures depicted, both on the floor of the mouth and in the area of the tongue, could be evaluated and interpreted well. Overall, there was a wide range of different image qualities and morphological conditions, from a rather “blurred, cloudy appearance” to clearly and distinctly visible cell structures (see Figs. 5 and 6).

## Discussion

In this study we have shown that CLE is suitable for imaging the surface condition of the mucosa within the entire oral cavity, although there are clear differences between the individual localizations (in terms of image quality and informative value).

It is already known from clinical examinations and previous anatomical knowledge that the mucosa of the oral cavity is not completely identical in its entirety at the various localizations. For example, there are clear differences in terms of vascular supply (several vascular plexi) and surface texture. There are keratinized and non-keratinized parts of the oral mucosa and specialized oral mucosa (such as taste buds and tonsils). Mechanically stressed areas of the oral mucosa (such as the hard palate and the attached gingiva in the alveolar ridge area) are keratinized to withstand the forces exerted on them (by food during chewing and swallowing). Non-keratinized areas (such as in the area of the floor of the mouth) have a rather low mechanical load and are called lining mucosa [15, 16]. All these anatomical differences are also likely to have an impact on the performance and interpretation of imaging with CLE.

To date, however, there are no studies that investigate these supposed influences or provide examples of the differences in CLE imaging in the various localizations of the oral cavity. In our opinion, however, this is a fundamental prerequisite for carrying out further examinations with CLE in the oral cavity and subsequently being able to make a sufficient distinction between physiological mucosa and benign or malignant mucosal changes.

Nathan et al. already mentioned in one of their preliminary studies [18] that different regions within the oral cavity are visualized better or worse. However, the individual localizations of the oral cavity were not systematically measured and there were no detailed descriptions or visual examples of how the individual regions of the oral cavity are visualized in CLE-imaging.

Our systematic CLE-examinations of the oral cavity show the morphologically most discriminative results in the area of the upper alveolar process as well as the palatal region (Fig. 2).

Especially the easy access and the osseous support of the fixed mucosa in the area of the alveolar ridge enabled a simple and sufficient CLE-Imaging.

In the area of the hard palate, sufficient image generation was more challenging due to the limited accessibility caused by the concave anatomy of the palate. Nevertheless, it was possible to obtain usable image material for each patient. In this area the tip of the probe sometimes could not be fully positioned on the tissue, so that parts of the generated image could not be used or analyzed. The parts of the images where the probe was fully positioned on the tissue were of good quality.

This corresponds to the statements of Nathan et al. This working group was also able to visualize the hard palate well with the help of CLE [18]. The area of the lips (intraoral) was not examined in their studies using CLE.

Within our study the best imaging results of all areas of the oral cavity that have no osseous support (such as the alveolar ridge and hard palate) were seen at intraoral labial site (Fig. 3). Overall, there was an increase in motion artifacts, a phenomenon which can be ascribed to the muscular support of the mucosa in this area. As an examiner, reliance on the patient’s compliance is evident. These motion artifacts can be clearly identified by (experienced) examiners and eliminated or not taken into account when viewing the image data. Initial studies on automatic evaluation of CLE images in the head and neck region have also shown that (motion) artifacts can already be automatically detected by a neural network [19, 20]. Overall, the images within our study showed an increased number of blood vessels in the lip mucosa. However, there were no malignant features of the vascular structure (like corkscrew-like vessels or dilated intraepithelial capillary loops) as described in a previous study by our research group [21]. This supports the assumption that these are physiological mucosal areas, consistent with the clinical appearance (even without histological confirmation).

The vessels appear bright white in confocal laser endomicroscopy due to the fluorescent dye they contain. For technical reasons (adaptive contrast/brightness setting of the CLE device), the other parts of the image seem therefore to be darker compared to the images of other localizations of the oral cavity. However, manually controlling the dynamic range compression (i.e., the scaling of the original sensor image to the displayed image), the surrounding tissue structure can be fully visualized.

Nathan et al. describe [18] that the subsites including the buccal mucosa, floor of the mouth and retromolar trigone can be visualized very well. This statement is not entirely consistent with our results. In the area of the buccal mucosa a sufficient image acquisition was not always possible without restrictions. The big muscular prevalence in this region together without any fixed mucosa sometimes disabled the examiner to gain an artifact-free scan. However, the examiner succeeded most of the time in the acquisition of a CLE image without any artifacts which was useable for further evaluation. In many CLE frames there are broad stripes or longitudinal oval shapes that appear to be horn pearls (see Fig. 4). This may be due to increased mechanical stress in the area of the intercalary line (due to friction on the teeth and bites).

Similar difficult circumstances for gaining informative CLE frames were given at the floor of mouth in sublingual region (Fig. 6). The main problem of receiving a good image was the permanent presence of saliva (with a patient in a sitting position) because of the low level of this region within the oral cavity and the local proximity of the excretory duct of the submandibular and sublingual salivary glands. Together with the lack of a fixed mucous membrane and the uneven surface, this made it considerably more difficult to produce reliable images. Most frames in this localization contained artifacts.

Some of the analyzable images showed the typical structures of physiological oral mucosa (e.g. homogeneous intercellular gaps and uniform cell shapes), others were rather “cloudy and blurred” (see Fig. 6).

Similar findings were observed in the area of the lateral edge of the tongue (Fig. 5). The CLE-scan in this area was associated with more difficulties because of the inability of the patient to keep the tongue absolutely relaxed together with its unique ability to move in every direction in space. Here we are even more dependent on patient compliance (to minimize motion artifacts). Many of the CLE images were only partially filled with the scanned region due to incomplete surface contact of the probe. The analyzable sequences also showed typical morphological structures, although the image quality exhibited a wide variation. Overall, it must be mentioned here that sufficient CLE-image generation and evaluation in the area of the lateral tongue margin was significantly more difficult in our study. This is not entirely consistent with the statements of Nathan et al. [18] who reported that visualization in the area of the dorsal tongue margin was difficult (due to the keratinized filiform papillae), but that the lateral and ventral tongue could be visualized well. In our investigations, imaging data generation was significantly more difficult in this region compared to the alveolar ridge and hard palate.

In addition to the given anatomical conditions, there are other factors that can influence and complicate CLE imaging. To date, many CLE-images have been acquired intraoperatively in patients under general anesthesia in preliminary studies. In this case, examiners are able to eliminate some difficulties which are added by an awake test person (such as increased movement artifacts, limited accessibility of the oral cavity or contamination of the probe by increased saliva).

In our opinion, this situation does not realistically reflect the clinical conditions in an (oral and maxillofacial) outpatient clinic, especially at the time of initial assessment of a suspicious oral mucosal lesion. A biopsy taken from an awake patient under local anesthesia is the clinically much more realistic and desirable condition, compared to an assessment under general anesthesia at first presentation.

Therefore, all images in our study were also taken from awake patients with underlying diseases from everyday clinical practice (chair-side). As already mentioned, it is not always easy to keep all scanned regions completely relaxed in an awake subject; the patient’s cooperation is an absolute prerequisite for reliable results.

In addition, it is more difficult to achieve a situation without saliva or blood in the oral cavity on an awake patient, especially in the sublingual cavity, which is a mandatory requirement for CLE-frames of sufficient quality.

Despite these difficult conditions, sufficient CLE image data could be collected over the entire oral cavity from (presumably) physiologic mucosa.

### Limitations of the study

It must be mentioned that the measured areas were not histopathologically examined and verified. Taking biopsies from clinically normal areas is not ethically justifiable and was therefore not carried out.

This implies that the cohort of patients examined is susceptible to a specific bias. All of them already presented themselves with a macroscopically suspicious lesion of their oral epithelium (which was examined and biopsied; not the subject of these evaluations), which implies a higher risk of further suspicious lesions within the oral cavity. Clinically unsuspicious areas were imaged and assessed as healthy oral mucosa but are always at risk of undetected changes in their cellular structure on CLE imaging. Another limitation of the methodology of pCLE is a limited penetration depth (of maximum 65 μm) that only enables imaging of superficial mucosa. Submucosal lesion cannot be detected and monitored over time (as already described in our previous publication [12]). In addition to the limited availability of CLE devices, there are other known technical limitations, such as the need for fluorescein (with the potential risk of an allergic reaction), which are not necessary with other optical methods such as high-definition ultrasound and OCT [22, 23].

All three methods mentioned (CLE, hd ultrasound and OCT) are optical “real-time” diagnostic procedures that have specific advantages and disadvantages. HD ultrasound and OCT are completely non-invasive methods. CLE is considered minimally invasive due to the fluorescein required. As described above, CLE has a technical limitation in terms of penetration depth. The generated image shows the tissue surface parallel to the probe surface and thus to the surface of the tissue to be examined. In contrast, tissue examination with hd ultrasound or OCT enables a cross-sectional evaluation in which the penetration depth into deeper tissue layers can also be assessed. This appears to be an advantage of these optical procedures. However, with hd ultrasound, for example, the transducer is significantly larger than a CLE probe (up to several centimeters vs. 2.8 mm), which makes access to anatomically difficult structures considerably more difficult. For this reason, studies on ultrasound examinations within the oral cavity have often been carried out in the area of the tongue [22]. The decisive advantage of CLE is the much higher magnification of the superficial mucous membranes (up to 1000-fold), which allows individual cells, the cell network and vessels to be visualized on a microscopic level.

All optical methods are currently additive measures for histopathological tissue examination (with necessary invasive tissue biopsies), which provide promising results and could possibly provide similarly meaningful results in the future. The exact advantages and disadvantages of the confocal laser endomicroscopy used in this study compared to histological examination (with the risk of injury to anatomical structures and infection) and other optical methods have already been discussed in several previous studies [12, 13, 17, 19–21, 24, 25] and will not be addressed here for reasons of repetition.

**Fig. 7:**
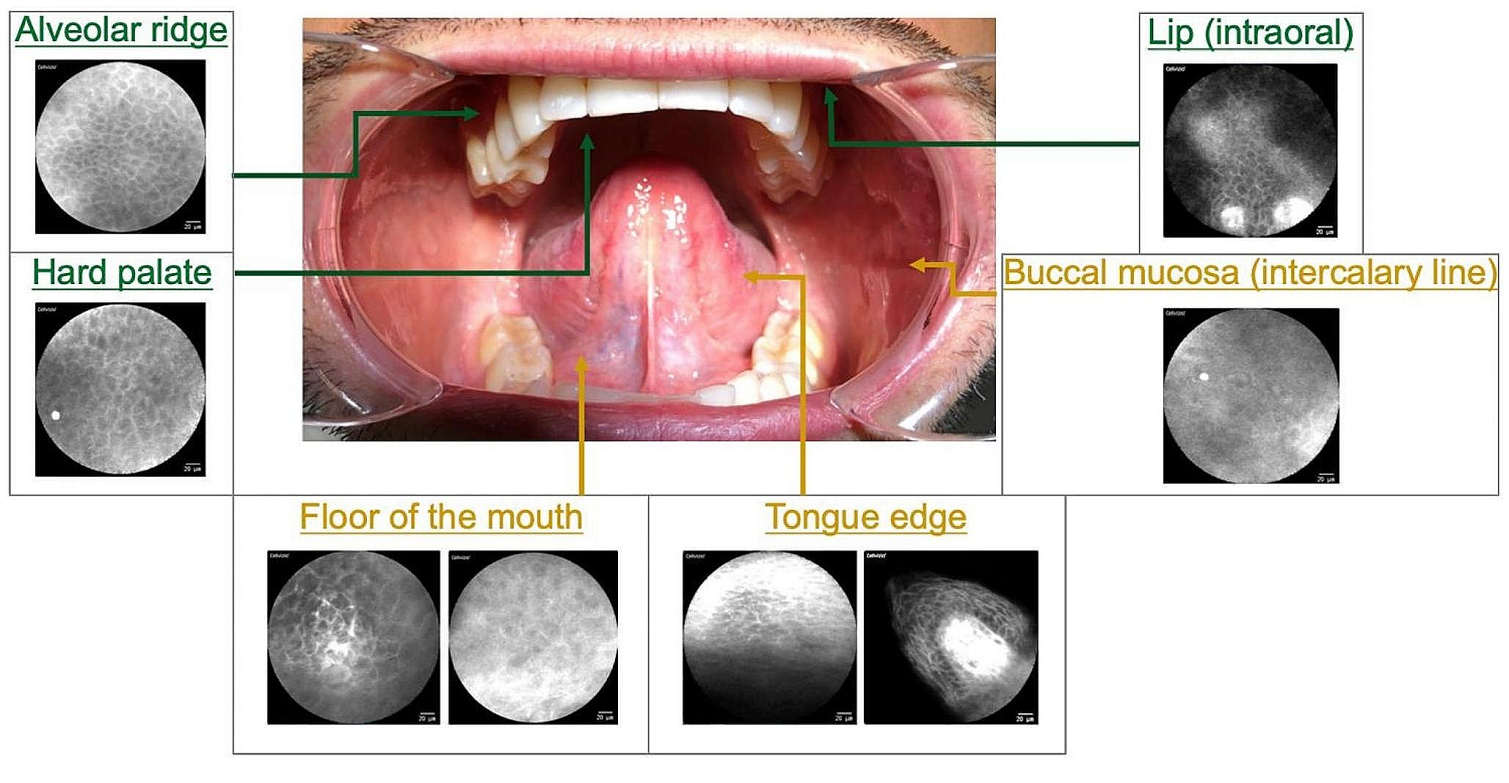
Overview of the CLE quality of image generation, visualization and usability within the oral cavity (physiological mucosa): Very good conditions for CLE diagnostics in the area of the alveolar ridge, the hard palate and the lip (*shown in green*); more difficult conditions in the rest of the oral cavity (due to increased artifacts, *shown in yellow*)

We assert that recording CLE frames is a valid method to obtain live images of epithelial architecture within the oral cavity. Identified factors influencing image quality and usability are the anatomical access to the region of interest, patient compliance and the amount of mucus/saliva. In addition, the heterogeneous architecture of the oral mucosa affects the results and therefore the practicability of CLE-imaging in a relevant way. It could be shown that regions with osseous support and fixed mucosa (attached gingiva) seem to be easier to scan than regions with flexible oral mucosa (lining mucosa) (Fig. 7).

Especially at the lateral border of the tongue and at the floor of the mouth it was more difficult to gain a suitable visualization of the epithelial architecture. Particularly at these sites of the oral cavity many HNSCCs are located [26].

One could argue that this is a notable drawback of the CLE method for an early detection of oral mucosal diseases. However, it is crucial to note that CLE images of histologically confirmed squamous cell carcinomas of this area, i.e., edge of the tongue and floor of the mouth, differ significantly from images of physiological mucosa (after a brief review of the new data as well as taking into account the previous publications on this subject [12, 14, 18]). However, this is not subject of the present study and requires further investigation; more detailed analyses and in particular statistical evaluations need to be carried out in this regard. The aim of this study was to determine whether CLE is suitable for diagnosis in the different areas of the oral cavity and what the different areas look like “physiologically”. From the authors’ point of view, this is the necessary first step and forms the basis for drawing meaningful conclusions in comparison with mucosal changes and being able to differentiate carcinomas reliably and validly in the long term.

## Conclusions

In summary, CLE can be utilized as a reproducible and non-invasive optical method for examining the oral cavity mucosa. In this study, for the first time in this field, different localizations within the oral cavity were systematically examined, visualized, and compared with each other by using CLE. It was shown that CLE imaging cannot be performed and interpreted equally in all areas of the mouth (concerning physiological mucosa).

The knowledge presented here forms the basis for all further CLE examinations in the oral cavity and can therefore improve the ability to discriminate physiological patterns from pathological conditions in the future while taking into account the localization in the oral cavity.

### Electronic supplementary material

Below is the link to the electronic supplementary material.

Supplementary Material 1

## Data Availability

No datasets were generated or analysed during the current study.
