# Supplementary material for: Oral mucosa - an examination map for confocal laser endomicroscopy within the oral cavity: an experimental clinical study
Source: Clin Oral Investig. 2024 Apr 23;28(5):266. doi: 10.1007/s00784-024-05664-9 (PMC11039507; doi:10.1007/s00784-024-05664-9)
Supplement: Supplementary file 1 — Supplementary Material 1 [file 784_2024_5664_MOESM1_ESM.pdf]

CLE Protocol

Measurement performed on:__________________

Patient name:____________________________ Date of birth: ___________________________

Smoker: ☐ n ☐ y, ___PY

renal retention parameters: ☐ inconspicuous ☐ increased

Allergies: ☐ n ☐ y, following: ________________________

β-blocker: ☐ n ☐ y

Localization of the lesion: [ ]

Known precursor lesion/
Oral mucosal disease : [ ]

- Macroscopic images/photos: Nr. : _______________

- CLE with contrast agent:

Fluorescein: **3ml**

	Sequences (after X mins)
1. labial region	
2. upper alveolar process	
3. lateral border of the tongue	
4. floor of the mouth	
5. hard palate	
6. buccal site (intercalary line)	
7. suspicious mucosal lesion	

Examiner : _____________________
